# Evaluation of Free Radical Scavenging and Antimicrobial Activity of Coleus amboinicus-Mediated Iron Oxide Nanoparticles

**DOI:** 10.7759/cureus.55472

**Published:** 2024-03-04

**Authors:** Faris Kunjan, Rajeshkumar Shanmugam, Sulochana Govindharaj

**Affiliations:** 1 Nanobiomedicine Lab, Centre for Global Health Research, Saveetha Medical College and Hospitals, Saveetha Institute of Medical and Technical Sciences, Chennai, IND

**Keywords:** eco-friendly method, product development, green synthesis, iron oxide nanoparticles, coleus amboinicus

## Abstract

Background

In this research, iron oxide nanoparticles were synthesized using *Coleus amboinicus *stem extract, which is used for various diseases such as throat infection, cough, fever, nasal congestion, and digestive problems.

Aim

This study aimed to formulate a green synthesis of iron oxide nanoparticles mediated by *Coleus amboinicus *(known as karpuravalli in Tamil) and assess its antimicrobial and antioxidant properties.

Materials and methods

Iron oxide nanoparticles were synthesized, and then their antimicrobial properties were tested against two specific pathogens, i.e., *Streptococcus mutans* and *Candida albicans,* using the agar well diffusion technique. The 2,2-diphenyl-1-picrylhydrazyl (DPPH) assay, hydroxyl radical scavenging (H_2_O_2_) assay, and ferric ion reducing antioxidant power (FRAP) assay were conducted to check the free radical scavenging activity.

Result

The results obtained showed that these iron oxide nanoparticles showed better antimicrobial activity against *Streptococcus mutans* when compared to *Candida albicans, *and the antioxidant activity showed a very close efficacy when compared to the standard.

Conclusion

The research has demonstrated the high antioxidant activity and high antibacterial activity of iron oxide nanoparticles using *Coleus amboinicus *stem*, *a* *natural and cheaper antimicrobial drug compared to the drugs present on the market.

## Introduction

*Coleus amboinicus Lour.*, often known as Lamiaceae, is a perennial plant native to Indonesia that is commonly grown in tropical Asia, Australia, and Africa. Its scientific name is *Plectranthus amboinicus *(Lour.) Spreng. Along with being utilized in folk medicine, *C. amboinicus* is also used as a spice and ornamental plant [1]. The medical efficacy of these plants comes from a class of active compounds known as phytochemicals, which have a clear physiological effect on the human body. The World Health Organization (WHO) states that the ideal source for obtaining a range of pharmaceuticals would be medicinal plants. Therapeutic treatments can greatly benefit from the use of plant extracts and phytochemicals with recognized antibacterial activities [2]. 

The biosynthetic process of nanoparticle formation may eliminate the need for chemicals and increase the biocompatibility of the nanoparticles. In the study and advancement of materials science and technology, the green synthesis technique is an effective synthesis process [3]. The reduction of waste, the use of safer solvents and renewable feedstock, as well as the reduction of pollutant derivatives, are some fundamental concepts of "green synthesis" [4]. Metal oxide nanoparticles have been identified as a promising alternative when addressing microbial resistance to antibiotics since they have lower toxicity, higher stability, more selectivity, and greater endurance than organic nanomaterials [5]. Because iron oxide nanoparticles are biocompatible, ecologically safe, and chemically and physically potent, they provide special qualities for therapeutic applications [6]. Iron oxide nanoparticles have been used in various fields like photocatalysis, biotechnology, environmental science, and medicine [7]. Iron oxide nanoparticles were commonly used and had the main applications for eliminating heavy metals, antibiotics, and dyes from water sources in the biomedical field of applications such as harmful to cancer cells and drug delivery in the target area [8]. 

Since synthetic antioxidants and antimicrobials have been demonstrated to have negative side effects, there is a need for natural antioxidants and antimicrobials that are more potent, less toxic, and more affordable [9]. Traditional antibacterial agents have been made from plant extracts. In contrast, metal oxide nanoparticles have lately attracted attention as potential antibacterial agents because of their straightforward and low-cost manufacturing processes [10]. The objective of the current research work was to determine the efficacy of antimicrobial activity and free radical scavenging activity using *C. amboinicus* stem-mediated iron oxide nanoparticles. 

## Materials and methods

Preparation of *C. amboinicus* stem extract

The* C. amboinicus* stem was freshly collected from the nanoherbal garden at Saveetha Dental College and Hospital, Chennai, India. The stem was washed in running water and cut into small pieces. Two grams of *C. amboinicus* stem were weighed and crushed into a fine paste. The ground stem was mixed with 100 mL of distilled water, and *C. amboinicus* stem solution was prepared. It was kept in the heating mantle for 15-20 minutes at 55℃. The boiling solution was filtered using a muslin cloth, and the filtered extract was used for nanoparticle preparation.

Preparation of iron oxide nanoparticles

To begin with, 0.322 gm of iron chloride was weighed and mixed with 50 mL of distilled water. The iron chloride solution was mixed with 50 mL of *C. amboinicus* stem extract. The reaction mixture (iron chloride solutions + *C. amboinicus* stem extract) was kept in the magnetic stirrer at 650 rpm for 48 hours. It was taken in the UV-visible spectrophotometer readings and preliminarily confirmed for synthesized nanoparticle solutions. The synthesized iron oxide nanoparticle solutions were centrifuged at 8,000 rpm for 10 minutes. After that, the pellet was collected in the sterile centrifuge tube, and the pellet solutions were used for further biomedical applications.

Antimicrobial activity

Applying the agar well diffusion method, the antibacterial properties of green-synthesized iron oxide nanoparticles were assessed. Mueller-Hinton agar plates were prepared and autoclaved for 15 to 20 minutes at 121°C to sterilize them. After sterilization, the medium was transferred to the surface of a sterile Petri plate and left to cool at room temperature. Using sterile cotton swabs, the microbial suspension (*Streptococcus mutans*, *Candida albicans*) was equally spread across the agar plates. A clean polystyrene tip was used to divide the agar plates into wells with a diameter of 9 mm. Subsequently, various concentrations of iron oxide nanoparticles (25 µg/mL, 50 µg/mL, and 100 µg/mL) were loaded into the wells. The usual treatment was an antibiotic as standard, like fungi (fluconazole) or bacteria (amoxyrite). The plates were incubated for 24 hours at 37°C for bacterial cultures and 48 hours at room temperature for fungal cultures. To evaluate the antibacterial activity, the diameter of the inhibitory zone around the wells was measured. The zone of inhibition's diameter was measured in millimeters (mm), after which the zone of inhibition's calculation was completed.

Time-kill curve assay

The microbiological properties and concentration-dependent interaction between *C. amboinicus-*mediated iron oxide nanoparticles and the ultimate growth rate of *S. mutans* and *C. albicans* across regular time intervals were evaluated using a time-kill curve experiment. Time-kill curve analysis was performed after the two (*S. mutans* and *C. albicans*) oral pathogens were cultured in Mueller-Hinton broth supplemented with various levels of iron oxide nanoparticles (25 µg/mL, 50 µg/mL, and 100 µg/mL). Before the test, growth curves were performed following a four-hour (one to four hours) pre-incubation period in a medium containing no antimicrobial drugs to make sure all pathogens had reached a stable early-to-mid log phase. Next, at regular intervals, the percentage of dead cells was measured at a wavelength of 600 nm [11]. 

Antioxidant activity

2,2-Diphenyl-1-Picrylhydrazyl (DPPH) Assay

In methanol, a stock solution of 0.1 mM DPPH was made. The stock solution was diluted to a final concentration of 20 M in methanol to provide a new working solution for every experiment. In a 96-well plate, 200 mL of the DPPH working solution was mixed with various concentrations of the iron oxide nanoparticles produced by *C. amboinicus* (10, 20, 30, 40, and 50 µg/mL). The plate underwent a 30-minute, room-temperature incubation period in the dark. At 517 nm, the absorbance was measured with a microplate reader. Using the following formula, the proportion of DPPH-scavenging activity was determined: 

% DPPH Scavenging Activity = [(Acontrol - Asample) / Acontrol] × 100 

where A sample is the absorbance of the sample (DPPH solution containing green synthesized iron oxide nanoparticles), and A control is the absorbance of the control (DPPH solution without the sample). Methanol was used as a blank. Ascorbic acid at a concentration of 1 mg/mL made up the positive control group. 

Hydroxyl Radical Scavenging (H_2_O_2_) Assay

The antioxidant activity was assessed in this work utilizing the H_2_O_2_ assay proposed by Halliwell et al.. To prepare 1 mL of the reaction mixture, 100 µL of 28 mM 2-deoxy-2-ribose was added. Various amounts of iron oxide nanoparticles mediated by *C. amboinicus* (10-50 µg/mL) were added. 100 µL of ascorbic acid, 200 µL of ethylenediaminetetraacetic acid (EDTA), and 200 µL of 200 µm ferric chloride were also added. Following a one-hour incubation period at 37 °C, the optical density at 532 nm was determined in comparison to the blank solution. The positive control was vitamin E. 

Hydroxyl radical scavenging activity (%) = [(Ablank − Asample)/Ablank] × 100 

Where A sample is the absorbance of the reaction with the sample, and Ablank is the absorbance of the control reaction (without the sample).

Ferric Ion Reducing Antioxidant Power (FRAP) Assay

Ferric ion reducing antioxidant power reagents: To prepare an acetate buffer with a 300 mM pH of 3.6, the following were weighed: (a) 3.1 grams of sodium acetate trihydrate, 16 ml of glacial acetic acid, and 1 liter of distilled water; (b) 2, 4, 6-tripyridyl-s-triazine (TPTZ): 10 mM in 40 mM hydrogen chloride (HCl) (molecular weight (MW): 312.34) (MW: 36.46), and (c) ferric chloride hexahydrate (FeCl_3_.6H_2_O): 20 mM, MW: 270.30. The working FRAP reagent was made by combining items a, b, and c in a ratio of 10:1:1 just before testing. Iron(II) sulfate heptahydrate (FeSO_4_.7H_2_O): The reference range was 0.1 to 1.5 mM in methanol. 

Procedure: After adding 3.6 mL of FRAP solution and 0.4 mL of distilled water, the mixture was incubated for five minutes at 37°C. Following the addition of this solution, 80 mL of iron oxide nanoparticles were added, and the mixture was incubated for 10 minutes at 37°C. The absorbance of the reaction mixture was measured at 593 nm. The calibration curve was created using five concentrations of FeSO_4_.7H_2_O (0.1, 0.4, 0.8, 1, 1.12, and 1.5 mM), and the absorbance values were computed similarly to sample solutions.

## Results

Preparation of *C. amboinicus* stem-mediated iron oxide nanoparticles

**Figure 1:**
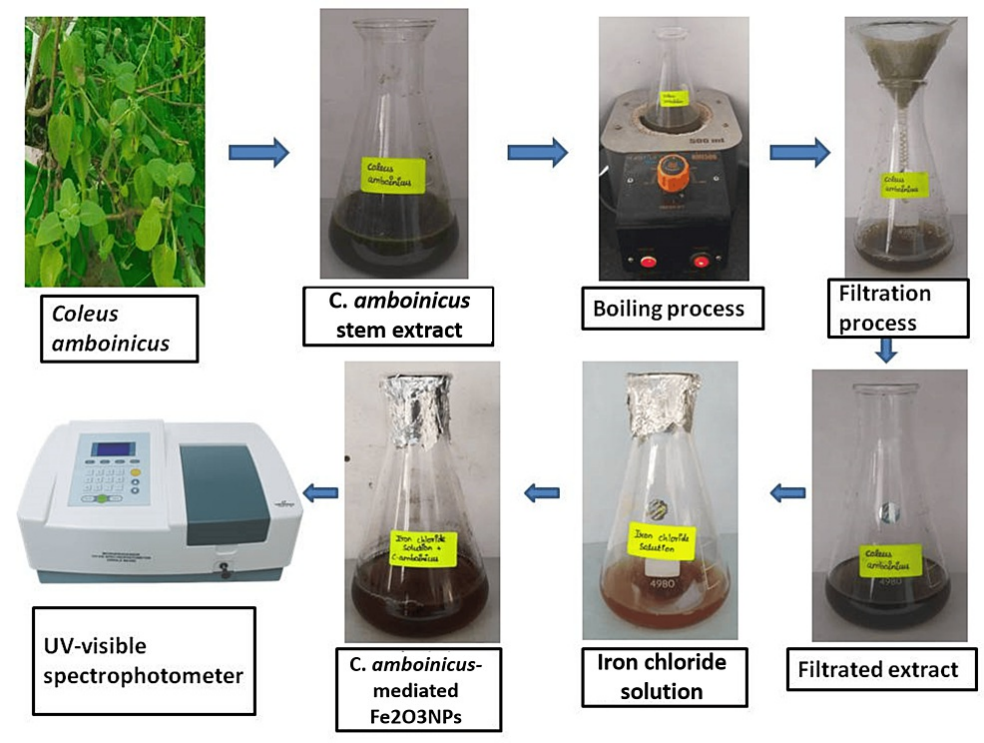
The green-synthesized iron oxide nanoparticles Fe_2_O_3_NPs: iron oxide nanoparticles; *C. amboinicus: Coleus amboinicus* *Coleus amboinicus *stem extract was used to synthesize the iron oxide nanoparticles

The iron oxide nanoparticles were green-synthesized using *C. amboinicus* stem extract, as shown in Figure 1. Iron chloride solution was used as the precursor, and *C. amboinicus *stem extract was used as a reducing agent. The combined solution was kept in the magnetic stirrer for 38 hours at 450 rpm. Ultraviolet-visible readings were taken for the prepared nanoparticles to preliminarily confirm the synthesized nanoparticles. The color of the nanoparticle solution was changed from yellowish brown to dark brown, and the precipitate was settled at the bottom of the conical flask. After the nanoparticle solution was centrifuged, the pellet was collected and stored in the refrigerator for further use.

Ultraviolet-visible spectroscopy analysis

**Figure 2:**
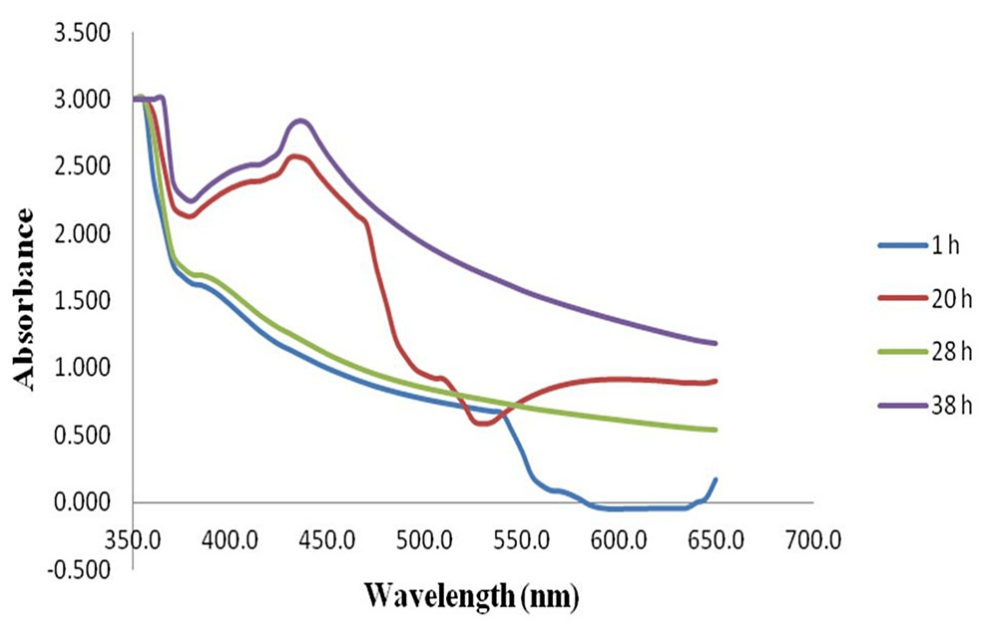
Ultraviolet-visible spectroscopy of the iron oxide nanoparticles Ultraviolet-visible spectroscopic readings for the prepared nanoparticles were taken at time intervals of one hour, 20 hours, 28 hours, and 38 hours. The UV spectrum revealed the presence of iron oxide nanoparticles in the maximum absorbent peak at 435 nm. h: hours

Ultraviolet-visible spectroscopy was used for the preliminary characterization of the synthesized nanoparticles. As shown in Figure 2, the absorbance of the prepared nanoparticle solution was measured using UV-visible spectroscopy, and the maximum peak was observed at 435 nm.

Antimicrobial activity

**Figure 3:**
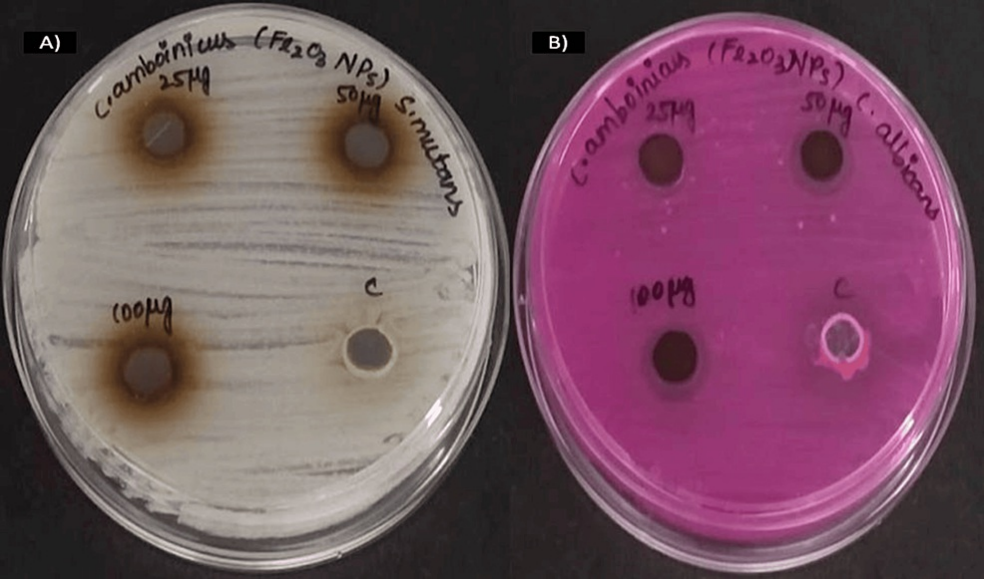
The agar well plate represents the antimicrobial activity. Iron oxide nanoparticles mediated *Coleus amboinicus *stem extract using the agar well diffusion technique. (A) *Streptococcus mutans; *(B) *Candida albicans* Fe_2_O_3_NPs: iron oxide nanoparticles; C: control (*Coleus amboinicus* stem extract); iron oxide nanoparticles used in different concentrations: 25µg, 50µg, and 100µg

**Figure 4:**
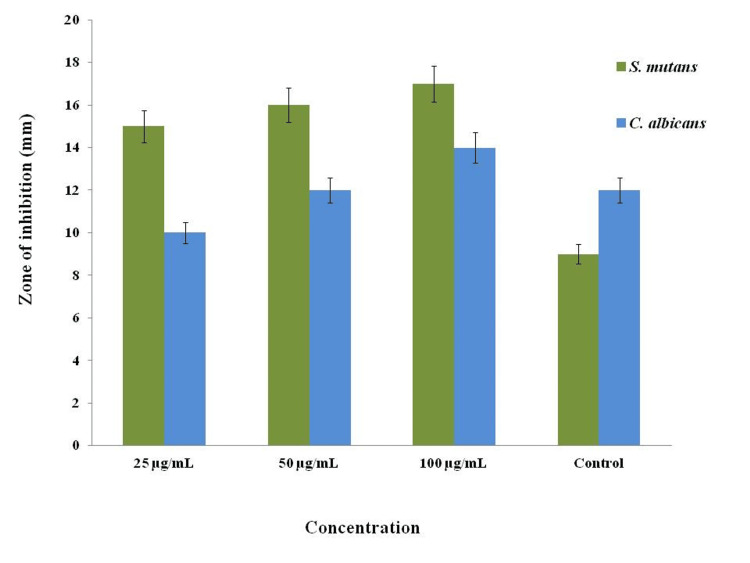
The graph represents the antimicrobial activity of the iron oxide nanoparticles. The antimicrobial efficacy of *Coleus amboinicus *stem-mediated iron oxide nanoparticles using oral pathogens, especially *Streptococcus mutans* and *Candida albicans​​​​​​​*

Antimicrobial activity was used to evaluate the iron oxide nanoparticles using *C. amboinicus* stem extract against oral pathogens such as *C. albicans* and *S. mutans* (Figure 3). The nanoparticles were added to three different concentrations of 25, 50, and 100 µg/mL in the agar well plates. In Figure 4, iron oxide nanoparticles mediated by *C. amboinicus* show the inhibitory zone against *C. albicans* and *S. mutans* at 14 mm and 17 mm for higher concentrations (100 µg/mL) and lower concentrations (25 µg/mL) shown at 10 mm and 15 mm, respectively. In comparison, the fourth well control contained commercial antibiotics and antifungal agents (amoxyrite and fluconazole) and showed an inhibitory zone of 9 mm against *S. mutans* and 12 mm against *C. albicans*. The nanoparticles showed better antimicrobial activity compared with commercial antibiotics and antifungal agents. 

Time-kill curve assay

**Figure 5:**
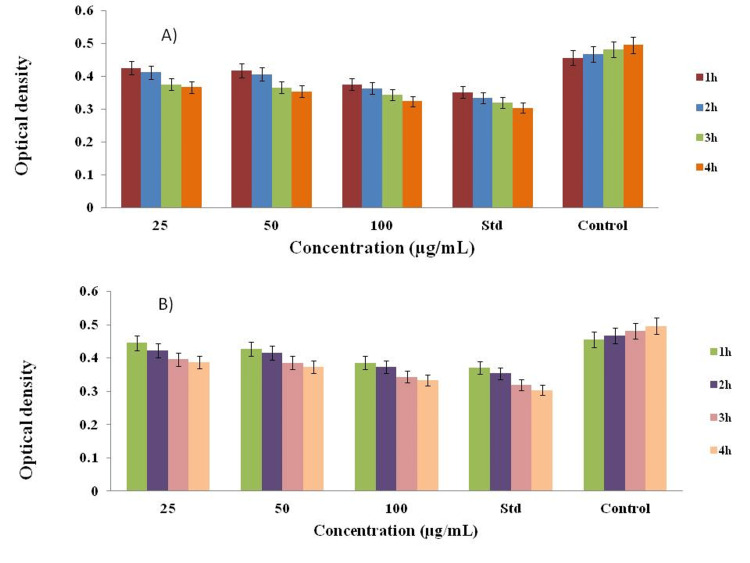
The graphs represent the time-kill curve assay Time-kill curve assay of *Coleus amboinicus* stem-mediated iron oxide nanoparticles; A) *Streptococcus*​​​​​​* mutans *and B) *Candida albicans* Std: standard (amoxyrite: *S. mutans* and fluconazole: *C. albicans* ); control: only microbial broth culture; h: hours

The time-kill curve assay was done for a duration of one hour to four hours. It demonstrated the time-kill curve assay of *C. amboinicus*-mediated iron oxide nanoparticles against *C. albicans*, as shown in Figure 5A. The optical density was measured in the enzyme-linked immunosorbent assay (ELISA) reader at 600 nm. The decrease in the number of *C. albicans* colonies was observed for some hours (one to four hours). When compared to the standard, the iron oxide nanoparticles at 100 μg/mL showed a higher reduction of fungal growth in the fourth hour. Higher fungal growth was found in the control compared with the standard solution and nanoparticles. Because the control, represented as the fresh fungal broth culture, was added, the standard representing the commercial antifungal agent (fluconazole) was added.

The time-kill curve assay was done for a duration of one hour to four hours. It demonstrated the time-kill curve assay for *C. amboinicus*-mediated iron oxide nanoparticles against *S. mutans* shown in Figure 5B. The optical density was measured in the ELISA reader at 600 nm. The decrease in the number of *S. mutans* colonies was observed for some hours (one to four hours). When compared to the standard, the iron oxide nanoparticles at 100 μg/mL showed a higher reduction of bacterial growth in the fourth hour. Higher bacterial growth was found in the control compared with the standard solution and nanoparticles. Because the control, represented by the fresh bacterial broth culture, was added, the standard representing the commercial antibiotics (amoxyrite) was added.

Antioxidant activity

**Figure 6:**
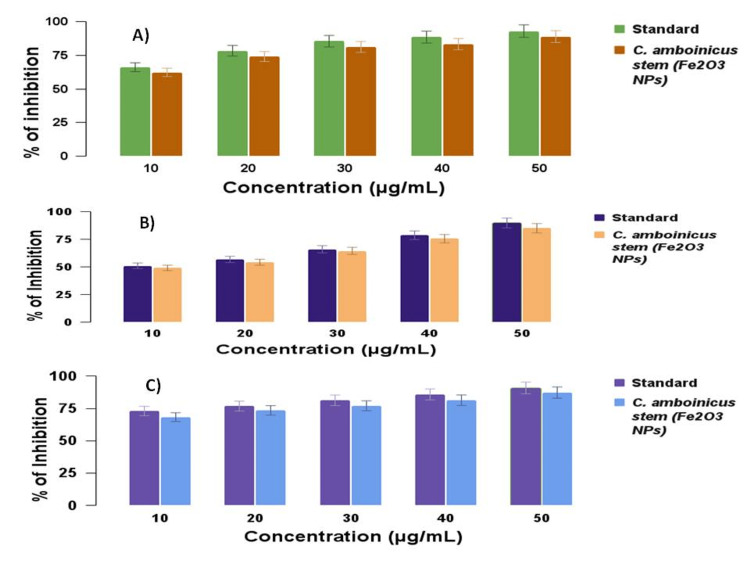
Graphical representation of the free radical scavenging activity of iron oxide nanoparticles mediated by Coleus amboinicus stem extract A) 2,2-diphenyl-1-picrylhydrazyl (DPPH) assay; B) hydroxyl radical scavenging (H_2_O_2_) assay; C) ferric ion reducing antioxidant power (FRAP) assay Standard: ascorbic acid (DPPH and FRAP assay), vitamin C for H_2_O_2_ assay Fe_2_O_3_NPs: iron oxide nanoparticles; %: percentage

The antioxidant activity was done using the DPPH technique. The DPPH assay was compared with standard ascorbic acid. The iron oxide nanoparticles have five concentrations, such as 10-50 μg/ml, which were added. The various (five) concentrations of iron oxide nanoparticles shown in Figure 6A indicate the percentage of inhibitions, such as 10 μg/ml having 65.8% inhibition, 20 μg/ml having 74.5% inhibition, 30 μg/ml having 78.3% inhibition, 40 μg/ml having 84.6% inhibition, and 50 μg/ml having 89.7% inhibition. The free radical scavenging activity of iron oxide nanoparticles using *C. amboinicus* stem extract was slightly similar when compared with the standard (66.23%-93.15%) inhibition. The standard represents ascorbic acid.

The antioxidant activity was done using the H_2_O_2_ technique. The H_2_O_2_ assay was compared with standard ascorbic acid. The iron oxide nanoparticles have five concentrations, such as 10-50 μg/ml, which were added. The various (five) concentrations of iron oxide nanoparticles shown in Figure 6B indicate the percentage of inhibitions, such as 10 μg/ml having 47.2% inhibition, 20 μg/ml having 54.1% inhibition, 30 μg/ml having 63.7% inhibition, 40 μg/ml having 72.9% inhibition, and 50 μg/ml having 78.6% inhibition. The free radical scavenging activity of iron oxide nanoparticles using *C. amboinicus* stem extract was slightly similar when compared with the standard for 51.1%-89.9% inhibition. The standard represents vitamin C.

The antioxidant activity was done using the FRAP technique. The FRAP assay was compared with standard ascorbic acid. The iron oxide nanoparticles have five concentrations, such as 10-50 μg/ml, which were added. In the various (five) concentrations of iron oxide nanoparticles shown in Figure 6C, the percentage of inhibitions such as 10 μg/ml had 66.4% inhibition, 20 μg/ml had 72.5% inhibition, 30 μg/ml had 80.1% inhibition, 40 μg/ml had 88.3% inhibition, and 50 μg/ml had 90.7% inhibition. The free radical scavenging activity of iron oxide nanoparticles using *C. amboinicus* stem extract was slightly similar when compared with the standard (72.98%-90.89%) inhibition. The standard represents ascorbic acid.

## Discussion

The current research work focuses on determining the potential efficacy of the antimicrobial and antioxidant effects of iron oxide nanoparticles synthesized by using the biological process catalyzed by *C. ambonicus* stem extract. Green-synthesized iron oxide nanoparticles using *C. amboinicus* stem extract were characterized by UV-visible spectrophotometry, visual observation, and in vitro studies (antimicrobial and antioxidant activity). The visual observation of iron oxide nanoparticles shows that the initial color was yellowish brown and the final stage color was dark brown, which represents the synthesized nanoparticles (Figure 1). In previous research, it was shown that the metal nanoparticle-mediated *Platanus orientalis* leaf extract has a reducing agent, and the color transformation was observed from yellowish brown to brownish black. The color changes were one of the most significant confirmations of the iron oxide nanoparticles synthesized [12]. After that, the maximum absorbent peak was observed in the preliminary characterization of UV-visible spectroscopy of iron oxide nanoparticles. Similarly, the iron oxide nanoparticle-mediated *Spirulina platensis* microalgae indicate a shift in the ferric (III) chloride's frequent absorption peak from 360 to 405 nm [13, 14]. The green-synthesized iron oxide nanoparticles were initially confirmed using a UV-visible spectrophotometer and tested for biomedical applications.

The antimicrobial activity of iron oxide nanoparticles against oral pathogens such as *S. mutans* and *C. albicans* was observed in both the agar well diffusion technique and the time-kill curve assay. Iron oxide nanoparticles demonstrate antimicrobial activity depending on the various concentrations. A higher concentration of nanoparticle solutions showed a reduction in the microbial growth count. Nanoparticles are small in size, which helps them enter the microbial cell wall to release the iron(II) (Fe2+) ions, and these ions have the ability to lyse the cell wall of the microbes, resulting in cell death. In the previous research work, Lagenaria siceraria leaf-mediated iron oxide nanoparticles had antimicrobial activity against *Staphylococcus aureus* and *Escherichia coli*. The maximum zone of inhibition was observed in *S. aureus* (8 mm) and *E. coli *(10 mm) [15]. Similarly, the metal nanoparticles showed antimicrobial activity against oral pathogens at a concentration of 500 µg/mL. The nanoparticles displayed an 18-mm inhibitory zone against *C. albicans* and a 16-mm inhibitory zone against *S. mutans* [16]. In previous research, iron oxide nanoparticles-mediated *Moringa oleifera* to determine the antimicrobial activity against human pathogens such as *S. mutans* (3.9+0.65), *S. aureus* (3.7 ± 0.67), *E. coli* (4.4 ± 0.42), and *Klebsiella sp.* (5.5 ± 0.50) [17]. In a previous research study to evaluate the time-kill curve assay for the effectiveness of *Terminalia chebula*-mediated metal oxide nanoparticles against wound pathogens, maximum bactericidal activity was shown at the highest concentration (1000 µg/mL) in the fifth hour [18].

Another biomedical application, such as antioxidant activity, was utilized to assess the free radical scavenging ability of the synthesized nanoparticles. The results revealed that the iron oxide nanoparticles, mediated by *C. amboinicus* stem extract, hold the potential for delivering antioxidant characteristics. The inhibition percentages, ranging from 65.8% to 89.7% in the DPPH assay and 47.2 to 78.6% in the H_2_O_2_ assay, exhibited increasing percentages of inhibition at higher concentrations (10 to 50 µg/mL). In related studies, metal oxide nanoparticles mediated by herbal formulations of *Matricaria recutita* and *Camellia sinensis* were employed as antioxidant-rich medications, showing inhibition percentages of 52%-87% in the DPPH assay and 48.2%-78.9% in the H_2_O_2_ assay [19]. Previous research involving *Borassus flabellifer* seed coat-mediated iron oxide nanoparticles also demonstrated antioxidant activity through DPPH assays, H_2_O_2_ assays, and ORAC assays. The inhibition percentages of the nanoparticles increased with concentration [20]. In conclusion, iron oxide nanoparticles mediated by *C. amboinicus *stem extract exhibit antioxidant properties, as evidenced by their ability to scavenge free radicals and inhibit oxidative processes. Additionally, these nanoparticles demonstrate antimicrobial properties against oral pathogens, causing microbial cell lysis and subsequent cell death. The use of nanoparticle solutions in the future is indicated for their safety and eco-friendliness.

Limitations

The study presents valuable insights into the antibacterial and antioxidant properties of *C. amboinicus* stem-mediated iron oxide nanoparticles, despite some limitations. However, assessing the durability and toxicity of these nanoparticles is imperative for potential therapeutic applications. The focus on short-term observations in the research poses challenges to fully understanding the stability and long-term impacts of the nanoparticles.

## Conclusions

In summary, the biologically mediated synthesis of iron oxide nanoparticles using the stem extract from *C. amboinicus *exhibits remarkable antimicrobial and antioxidant properties. The green synthesis method is both biocompatible and environmentally friendly, offering a cost-effective approach. The antioxidant activity of iron oxide nanoparticles demonstrates a dose-dependent effect, displaying their potential as an alternative to standard antioxidants. Further research is essential to understand the effectiveness of these novel medications in a clinical setting for treating oral lesions caused by oxidative stress and free radicals.
